# Lung Function Changes and Connective Tissue Growth Factor Expression in Idiopathic Pulmonary Fibrosis, Other Progressive Fibrosing Interstitial Lung Diseases, and Post-COVID Fibrosis

**DOI:** 10.7759/cureus.88645

**Published:** 2025-07-24

**Authors:** Elene Sherozia, Clive Page, Tamuna Kakhniashvili, Nino Tabagari, Maia Gotua

**Affiliations:** 1 Internal Medicine, David Tvildiani Medical University, Tbilisi, GEO; 2 Internal Medicine, The Center for Diabetes, Endocrine and Cardio-Pulmonary Diseases Diacor, Tbilisi, GEO; 3 Pharmacology, Sackler Institute of Pulmonary Pharmacology, King’s College, London, GBR; 4 Diabetes and Endocrinology, The Center for Diabetes, Endocrine and Cardio-Pulmonary Diseases Diacor, Tbilisi, GEO; 5 Allergy and Immunology, David Tvildiani Medical University, Tbilisi, GEO; 6 Allergy and Immunology, Center of Allergy and Immunology, Tbilisi, GEO

**Keywords:** connective tissue growth factor (ctgf), diffuse lung capacity for carbon monoxide (dlco), forced vital capacity (fvc), idiopathic pulmonary fibrosis (ipf), interstitial lung diseases (ilds), post-covid-19, progressive fibrosing interstitial lung diseases (f-ilds), progressive pulmonary fibrosis

## Abstract

Background

The clinical course of patients with idiopathic pulmonary fibrosis (IPF) and other progressive interstitial lung diseases (F-ILDs) varies from mild to severe worsening, which makes the development of new diagnostic and prognostic methods even more urgent. A number of studies have shown that plasma connective tissue growth factor (CTGF) is elevated during IPF, and that the levels of this substance are correlated with the changes in forced vital capacity (FVC).

The aim of our study was to investigate CTGF levels in patients with F-ILDs group, including its subgroups IPF and other F-ILDs, as well as post-COVID-19 cases, and to assess their association with lung function changes over a 12-month period.

Methods

A prospective cohort study was conducted with patients observed over 30 months. The involvement period was 18 months, followed by a 12-month observation period.

A total of 86 subjects were enrolled in the study. FVC (measured by spirometry), diffusing lung capacity for carbon monoxide (DLCO), and CTGF levels in blood serum (measured by enzyme-linked immunosorbent assay (ELISA)) were assessed at the beginning and end of the study.

Results

Regression analysis of the correlations between mean serum CTGF levels, FVC, and DLCO changes in the F-ILDs group demonstrated a significant negative correlation between the changes in mean serum CTGF levels, FVC, and DLCO. Similarly, in the IPF, idiopathic nonspecific interstitial pneumonia (iNSIP) and chronic sarcoidosis (IV stage) subgroups, there was a significant negative correlation between changes in mean serum CTGF levels, FVC, and DLCO. However, in the post-COVID-19 group, regression analysis did not reveal any correlations between changes in mean serum CTGF concentrations, FVC, and DLCO.

Conclusion

Our data suggest a correlation between decreased pulmonary function and increased CTGF levels in patients with IPF and other PPF conditions. Therefore, CTGF emerges as a potential surrogate marker for fibrosis progression in these patients.

In contrast, post-COVID-19 fibrosis appears to be a non-progressive fibrotic disease; however, further monitoring is necessary.

## Introduction

Interstitial lung diseases (ILDs) encompasses a broad and heterogeneous group of pulmonary parenchymal disorders characterized by similar clinical presentations and intraparenchymal fibrotic lesions, confirmed via high-resolution computed tomography (HRCT) [1-4]. Early and accurate diagnosis of ILDs remains challenging due to nonspecific symptoms, making the assessment of disease progression more difficult. ILDs include a group of diseases with both known and unknown causes, and the most common disease of unknown etiology is idiopathic pulmonary fibrosis (IPF) characterized by disease progression and has a poor prognosis despite management [2,3].

Patients with other types of fibrosing interstitial lung diseases are also at risk of developing a progressive fibrosis phenotype [2,3]. These include idiopathic nonspecific interstitial pneumonia (iNSIP), unclassified idiopathic interstitial pneumonia, interstitial lung disease associated with connective tissue diseases (CTD-ILD), chronic sarcoidosis (stage IV), fibrotic hypersensitivity pneumonitis (F-HP), and diseases related to occupational exposures, such as asbestosis and silicosis [2,3,5]. The term progressive pulmonary fibrosis (PPF) is used to describe a progressive disease course in persons with a fibrosing ILD other than IPF [5].

Some chronic interstitial lung diseases are drug-related or caused by viral infections [6-8]. Approximately 20% of patients with COVID-19 present with subpleural reticular changes, traction bronchiectasis and mild bilateral ground glass opacities (GGO), characteristic of pulmonary fibrosis [9,10]. Fibrosis progression has been observed in some patients, accompanied by radiological signs and measurable declines in lung function parameters, including forced vital capacity (FVC) and diffusing capacity for carbon monoxide (DLCO) [11-14].

The clinical course of progressive fibrosing interstitial lung diseases (PF-ILDs) [2] ranges from mild symptoms to severe and rapid deterioration. This variability highlights the urgent need for improved diagnostic and prognostic tools [15-19], particularly non-invasive biomarkers. Biomarkers can offer insight into disease activity and may help guide clinical decision-making.

Several candidates have been studied, including KL-6, a high-molecular-weight glycoprotein produced by regenerating type II pneumocytes. KL-6 levels are often elevated in fibrosing interstitial lung diseases, although their predictive value for disease progression remains uncertain [16].

Recent research has also turned attention to connective tissue growth factor (CTGF), a key mediator in fibrotic pathways. CTGF has been shown, in both in vitro and in vivo models, to be essential for the profibrotic activity of transforming growth factor-beta (TGF-β). It plays a central role in connective tissue formation and remodeling. In patients with IPF, elevated plasma CTGF levels have been found to correlate with changes in FVC, suggesting its potential as a biomarker of disease progression [20-24].

In this study, we investigated CTGF levels in patients with IPF, other PPF, and post-COVID-19 fibrosis and analyzed changes in lung function over a 12-month period.

## Materials and methods

A prospective cohort study was conducted, with patients observed over 30 months, including an 18-month involvement period and a 12-month observation period. Ethical approval was obtained from the clinic DIACOR and its independent ethics committee.

A total of 86 participants (41 men and 45 women; aged 40-85 years) were enrolled in the study. Among them, 48 adult patients (24 men and 24 women; aged 40-85 years) with PF-ILDs were included. Diagnoses were made according to the American Thoracic Society (ATS), European Respiratory Society (ERS), Japanese Respiratory Society (JRS), and Asociación Latinoamericana de Tórax (ALAT) guidelines (Raghu, 2018-2022), based on a ≥5% relative decline in FVC and a ≥10% relative decline in diffusing capacity of the lung for carbon monoxide (DLCO) in the previous 12 months with a combination of symptoms worsening. Predicted FVC percentage was ≥45%, and hemoglobin-adjusted DLCO was ≥30%.

From the PF-ILD group, 24 subjects who met the diagnostic criteria for IPF were identified and analyzed as the IPF subgroup, all of whom received pirfenidone background therapy (2403 mg daily dose for at least for the past 12 months). Additionally, 24 subjects with F-ILDs other than IPF, who had not received antifibrotic treatment, were included (all of them were under immunosuppression therapy). A further 26 adults with post-COVID-19 fibrosis (16 men and 10 women; aged 40-85 years) were enrolled. Finally, 12 healthy volunteers (one man and 11 women; aged 40-70 years) were included as controls. Interstitial pulmonary fibrosis was defined based on findings from HRCT scans. Patients without subpleural fibrosis on HRCT and those who had experienced a severe exacerbation of PPF or IPF within the last six months were excluded from the study.

Pulmonary Function Tests (PFTs): FVC (Spirometry; CareFusion, Numed Healthcare, Sheffield, UK) and DLCO (Body Plethysmography; MGC Diagnostics, St Paul, MN, USA) were assessed at the beginning and end of the study, alongside HRCT scans.

CTGF measurements

CTGF levels were measured in blood serum using an enzyme-linked immunosorbent assay (ELISA). Venous blood samples (2.5 mL) were drawn from an antecubital vein. Samples were allowed to clot for 10-20 minutes at room temperature and then centrifuged at 3000 rpm for 20 minutes. The supernatant was collected in aliquots and stored at -20°C for one month or at -80°C for up to six months. CTGF concentration was measured using the Human CTGF ELISA Kit (MyBioSource Inc., San Diego, CA, USA), with a normal range of 4.7-128.5 ng/L and a sensitivity of 10.29 ng/L.

Statistical analysis

Data were analyzed using analysis of variance (ANOVA) for group comparisons. Correlations were assessed using linear regression analysis. P-values <0.05 were considered statistically significant.

## Results

A total of 86 participants were enrolled in the study: 48 subjects in the PF-ILD group, 26 in the post-COVID-19 fibrosis group, and 12 healthy controls. Within the PF-ILD group, participants were further categorized into subgroups: 24 in the IPF subgroup, 10 in the iNSIP subgroup, seven in the connective tissue disease-related interstitial lung disease (CTD-ILD) subgroup, four in the sarcoidosis subgroup, and three in the silicosis subgroup.

**Figure 1:**
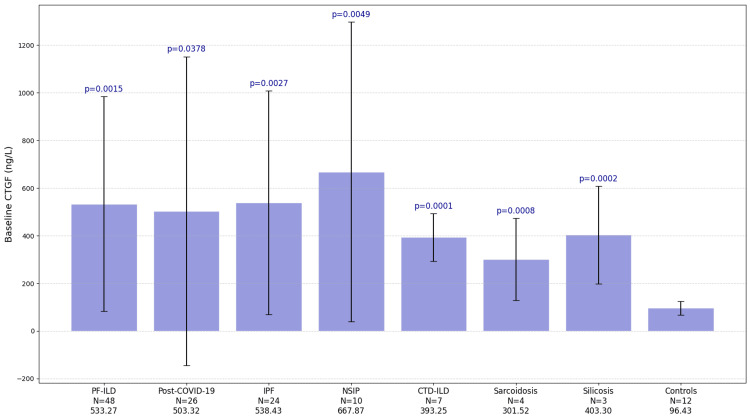
The baseline CTGF level was significantly higher in all groups compared to the healthy controls CTGF: Connective tissue growth factor; PF-ILD: progressive fibrosing interstitial lung diseases; IPF: idiopathic pulmonary fibrosis; NSIP: nonspecific interstitial pneumonia; CTD-ILD: connective tissue disease-related interstitial lung disease; FVC: forced vital capacity; DLCO: diffusing capacity for carbon monoxide.

Mean serum CTGF concentration at baseline was 533.27±451.03 ng/L in the PF-ILD group, 803.32±648.61 ng/L in the post-COVID-19 fibrosis group, and 96.43±28.98 ng/L in the control group. The mean serum CTGF concentrations in the subgroups were as follows: IPF, 538.43±469.82 ng/L; iNSIP, 667.87±629.05 ng/L; CTD-ILD, 393.25±100.27 ng/L; sarcoidosis, 301.52±171.98 ng/L; and silicosis, 403.30±204.40 ng/L (Figure 1).

The mean pulmonary function parameters in groups and subgroups at baseline were as follows. The mean FVC (% predicted) was 81.5±27.1 in the PF-ILD group and 72.2±23.2 in the post-COVID-19 fibrosis group. Subgroup values were: IPF, 83.1±23.4; iNSIP, 77.6±22.8; CTD-ILD, 85.8±14.3; sarcoidosis, 67.7±8.0; and silicosis, 64.6±6.0. The mean DLCO (% predicted) was 60.7±20.9 in the PF-ILD group and 54.0±15.2 in the post-COVID-19 fibrosis group. Subgroup values were: IPF, 62.8±32.3; iNSIP, 56.6±19.2; CTD-ILD, 76.5±15.0; sarcoidosis, 61.0±12.8; and silicosis, 63.3±2.6.

At the end of the study, the mean serum CTGF concentrations were 843.7±704.0 ng/L in the PF-ILD group and 978.4±944.3 ng/L in the post-COVID-19 fibrosis group. Subgroup values were: IPF, 927.3±560.9 ng/L; iNSIP, 1143.4±1115.1 ng/L; CTD-ILD, 560.9±284.5 ng/L; sarcoidosis, 471.4±194.4 ng/L; and silicosis, 516.3±125 ng/L.

The mean FVC (% predicted) at the end of the study was 64.7±13.6 in the PF-ILD group and 86.3±20.5 in the post-COVID-19 fibrosis group. Subgroup values were: IPF, 64.4±14.4; iNSIP, 60.8±16.1; CTD-ILD, 69.5±8.2; sarcoidosis, 73.5±17.0; and silicosis, 72.7±5.4. The mean DLCO (% predicted) at the end of the study was 50.6±15.7 in the PF-ILD group and 69.5±17.8 in the post-COVID-19 fibrosis group. Subgroup values were: IPF, 47.3±15.6; iNSIP, 43.6±12.9; CTD-ILD, 66.2±11.2; sarcoidosis, 57.5±8.8; and silicosis, 61.7±4.9 (Table 1).

**Table 1:** Baseline and 12-Month Mean Values of CTGF, FVC, and DLCO Across Groups PF-ILD: Progressive fibrosing interstitial lung diseases; CTGF: connective tissue growth factor; FVC: forced vital capacity; DLCO: diffusing capacity for carbon monoxide; IPF: idiopathic pulmonary fibrosis; NSIP: nonspecific interstitial pneumonia; CTD-ILD: interstitial lung disease associated with connective tissue diseases; SD: standard deviation.

Groups	Age (Mean±SD)	Baseline CTGF (Mean±SD)	Baseline FVC (Mean±SD)	Baseline DLCO (Mean±SD)	12-month CTGF (Mean±SD)	12- month FVC (Mean±SD)	12-month DLCO (Mean±SD)
PF-ILD (N=48)	66.9±8.4	533.27±451.03	81.5±27.1	60.7±20.9	843.7±704.0	64.7±13.6	50.6±15.7
Post-Covid (N=26)	62.8±11.2	503.32±648.61	72.2±23.2	54.0±15.2	978.4±944.3	86.3±20.5	69.5±17.8
IPF (N=24)	69.6±8.3	538.43±469.82	83.1±23.4	62.8±32.3	927.3±560.9	64.4±14.4	47.3±15.6
NSIP (N=10)	66.4±7.4	667.87±629.05	77.6±22.8	56.6±19.2	1143.4±1115.1	60.8±16.1	43.6±12.9
CTD-ILD (N=7)	57.8±6.0	393.25±100.27	85.8±14.3	76.5±15.0	560.9±284.5	69.5±8.2	66.2±11.2
Sarcoidosis (N=4)	69±1.6	301.52±171.98	67.7±8.0	61.0±12.8	471.4±194.4	73.5±17.0	57.5±8.8
Silicosis (N=3)	61.3±4.8	403.3±204.4	64.6±6.0	63.3±2.6	516.3±125.0	72.7±5.4	61.7±4.9
Controls (N=12)	53.5±8.0	96.43±28.98					

Mean serum CTGF concentrations at baseline were significantly higher in the PF-ILD and post-COVID-19 groups compared to healthy controls (Figure 1). F-statistics: PF-ILD, p=0.0015; post-COVID-19, p=0.0378. Subgroup analysis of mean CTGF levels showed the following F-statistics: IPF, p=0.0027; iNSIP, p=0.0049; CTD-ILD, p<0.0001; sarcoidosis, p=0.0008; and silicosis, p=0.0002.

Mean serum CTGF levels were significantly elevated at the 12-month follow-up visit in the PF-ILD group, with a mean change of 129.87%±182.29%, p=0.00427 (Table 2). In the IPF subgroup, mean serum CTGF levels were significantly higher compared to healthy controls, with a mean change of 161.57%±194.53%, p=0.01345. However, there was insufficient data to observe a significant increase in mean serum CTGF levels in the iNSIP, CTD-ILD, sarcoidosis, and silicosis subgroups.

**Table 2:** CTGF, FVC, DLCO Changes within a 12 months follow-up period in all groups CTGF: Connective tissue growth factor. PF-ILD: progressive fibrosing interstitial lung diseases; IPF: idiopathic pulmonary fibrosis; NSIP: nonspecific interstitial pneumonia; CTD-ILD: connective tissue disease-related interstitial lung disease; FVC: forced vital capacity; DLCO: diffuse lung capacity for carbon monoxide.

Data	Name	μ ∆ (%)	M ∆ (%)	Std Dev (%)	Min∆(%)	Max ∆ (%)	ANOVA F-stats	ANOVA p-value
PostCovid	CTGF	260.92	150.38	332.43	-45.36	1463.84	4.35	0.04206
PostCovid	FVC	26.37	22.44	31.45	-33.73	107.50	5.23	0.02652
PostCovid	DLCO	38.54	29.37	52.87	-40.68	155.17	10.99	0.00171
IPF	CTGF	161.57	96.54	194.53	-43.73	703.62	6.61	0.01345
IPF	FVC	-20.29	-21.85	11.45	-36.84	18.18	10.62	0.00210
IPF	DLCO	-18.28	-18.01	17.45	-65.12	15.38	4.31	0.04353
NSIP	CTGF	95.54	54.21	127.09	-22.48	425.18	1.27	0.27409
NSIP	FVC	-19.81	-21.19	13.32	-46.05	4.65	3.26	0.08763
NSIP	DLCO	-20.31	-20.69	17.85	-42.11	17.07	2.84	0.10942
Sarcoidosis	CTGF	161.82	12.14	320.81	-19.39	642.38	1.44	0.27464
Sarcoidosis	FVC	7.36	0.75	16.29	-3.64	31.58	0.28	0.61506
Sarcoidosis	DLCO	-3.97	-4.34	11.77	-14.71	7.50	0.15	0.71036
Silicosis	CTGF	113.71	-7.81	224.64	-24.00	372.93	0.44	0.54124
Silicosis	FVC	3.06	8.06	10.02	-8.47	9.59	0.07	0.80021
Silicosis	DLCO	-2.49	-8.20	10.55	-8.96	9.68	0.18	0.69433
CTD-ILD	CTGF	58.46	67.87	62.96	-54.16	129.39	3.16	0.10068
CTD-ILD	FVC	-17.89	-19.23	5.09	-25.23	-12.12	3.15	0.10125
CTD-ILD	DLCO	-13.23	-16.67	9.43	-23.53	2.70	2.22	0.16246

At the 12-month follow-up visit, mean serum CTGF levels were significantly elevated in the post-COVID-19 group, with a mean change of 260.92%±332.43%, p=0.04206 (Table 2).

FVC was significantly decreased at the 12-month follow-up visit in the PF-ILD group, with a mean change of -17.34% ± 14.86% (p=0.00026, Table 2). FVC was also significantly decreased in the IPF subgroup, with a mean change of -20.29%±11.453% (p=0.00210). However, no significant decrease in FVC was observed in the iNSIP, CTD-ILD, sarcoidosis, or silicosis subgroups. In contrast, FVC was significantly increased in the post-COVID-19 group, with a mean change of 26.37%±31.45% (p=0.02652, Table 2).

DLCO was significantly decreased at the 12-month follow-up visit in the PF-ILD group, with a mean change of -14.76%±13.99% (p=0.00987, Table 2). Similarly, DLCO decreased significantly in the IPF subgroup, with a mean change of -18.28%±17.45% (p=0.04353). However, no significant decrease in DLCO was observed in the iNSIP, CTD-ILD, sarcoidosis, or silicosis subgroups. In contrast, mean DLCO levels in the post-COVID-19 group were significantly increased, with a mean change of 38.54%±52.87% (p=0.00171, Table 2).

Regression analysis of the correlations between mean serum CTGF levels and changes in FVC in the PF-ILD group revealed a significant negative correlation. As mean serum CTGF levels increased, FVC tended to decrease (R²=0.06, p=0.00010). In the IPF, iNSIP, and sarcoidosis subgroups, a similar significant negative correlation was observed between changes in mean serum CTGF levels and FVC. As CTGF levels increased, FVC tended to decrease. The R² values were 0.12 for IPF (p=0.00088), 0.30 for iNSIP (p=0.00065), and 0.83 for sarcoidosis (p=0.01600), respectively.

**Figure 2:**
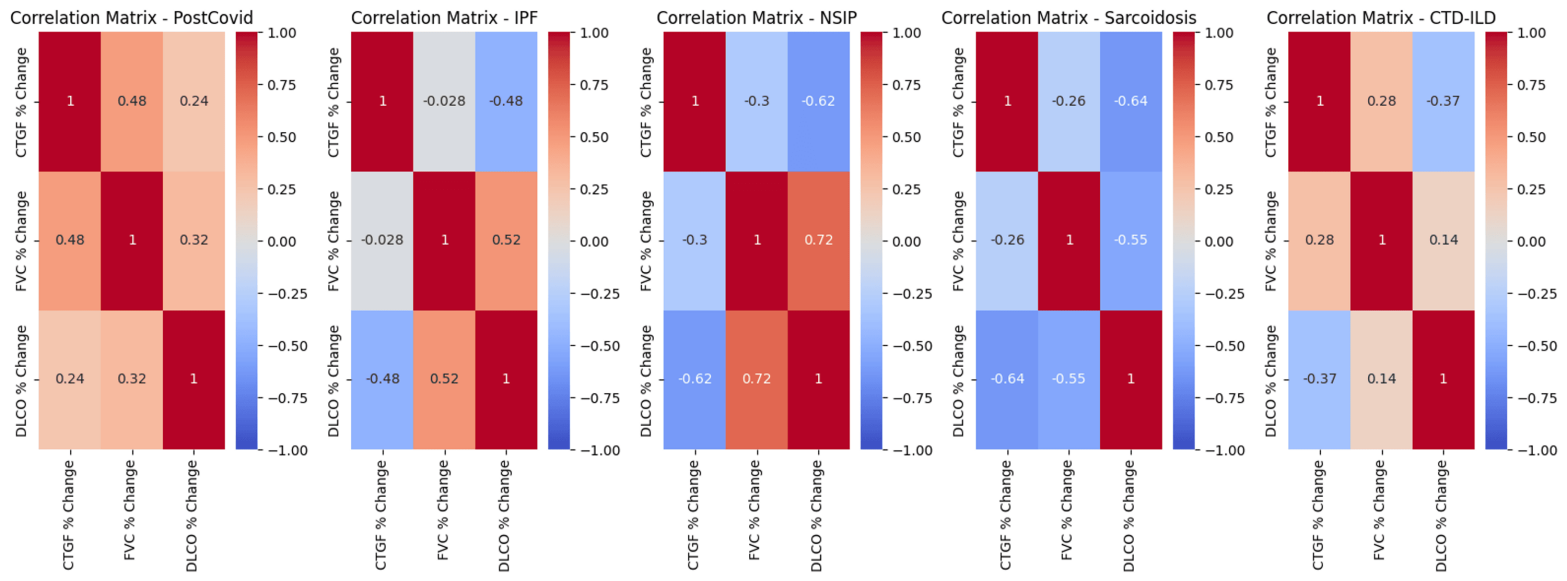
Correlation Matrix CTGF: Connective tissue growth factor; PF-ILD: progressive fibrosing interstitial lung diseases; IPF: idiopathic pulmonary fibrosis; NSIP: nonspecific interstitial pneumonia; CTD-ILD: connective tissue disease-related interstitial lung disease; FVC: forced vital capacity; DLCO: diffuse lung capacity for carbon monoxide.

No significant correlation was observed between mean changes in serum CTGF levels and FVC in the CTD-ILD or silicosis subgroups. The R² values were 0.08 (p=0.65932) and 0.35 (p=0.99556), respectively. Similarly, regression analysis in the post-COVID-19 group did not reveal any significant correlations, with an R² value of 0.23 and a p-value of 0.05837 (NS) (Figure 2).

Regression analysis of correlations between changes in mean serum CTGF concentrations and changes in DLCO in the PF-ILD group revealed a significant negative correlation. When CTGF levels increased, DLCO tended to decrease (R²=0.31, p<0.00001). In the IPF, iNSIP, CTD-ILD, and sarcoidosis subgroups, a similar significant negative correlation was observed. As CTGF levels increased, DLCO also decreased. The R² values were 0.12 for IPF (p=0.00088), 0.64 for iNSIP (p=0.00003), 0.93 for CTD-ILD (p=0.00027), and 0.74 for sarcoidosis (p=0.02796).

No significant correlation was found between changes in mean serum CTGF concentrations and changes in DLCO in the silicosis subgroup. The R² value was 0.19 (p=0.99708, NS) (Figure 2).

Regression analysis of correlations between changes in mean serum CTGF concentrations and changes in DLCO in the post-COVID-19 group did not reveal any significant correlations (R²=0.10, p=0.27459, NS).

Regression analysis of correlations between changes in FVC and changes in DLCO in the PF-ILD group revealed a substantial positive correlation between these two parameters. When FVC decreased, DLCO also tended to decrease (R²=0.35, p<0.00001). In the IPF and iNSIP subgroups (Figure 2), a similar positive correlation was observed. The R² for IPF was 0.29 (p=0.00103), while for iNSIP, R²=0.66 (p=0.00023).

However, no significant correlations were found between changes in FVC and changes in DLCO in the CTD-ILD, sarcoidosis, or silicosis subgroups. The R² values and corresponding p-values were as follows: CTD-ILD, R²=0.02 (p=0.69450, NS); sarcoidosis, R²=0.31 (p=0.44561, NS); and silicosis, R²=0.22 (p=0.92952, NS).

In the post-COVID-19 group, regression analysis of correlations between changes in FVC and changes in DLCO was also not statistically significant (R²=0.10, p=0.11453, NS) (Figure 2).

## Discussion

Our study demonstrated that serum CTGF levels were significantly higher in all patients with PF-ILD (IPF + F-ILDs) and in the post-COVID-19 group compared to healthy controls. Across all subgroups, CTGF serum levels were elevated relative to controls. In PF-ILD (IPF+F-ILDs) patients, CTGF serum levels showed a significant increase from baseline to the 12-month follow-up, predominantly driven by patients in the IPF subgroup, as no significant changes were observed in the other subgroups (iNSIP, CTD-ILD, sarcoidosis, or silicosis).

Regression analysis revealed significant negative correlations between CTGF levels and pulmonary function tests in patients with IPF, iNSIP, CTD-ILD, and sarcoidosis. Increased serum CTGF levels were associated with reduced FVC and/or DLCO in patients with PPF and in the IPF, iNSIP, CTD-ILD, and sarcoidosis subgroups.

Previous studies have similarly reported elevated CTGF (CNN2) levels correlating with declining pulmonary function in IPF patients [10,11]. However, this is the first study to examine the most PF-ILD (IPF+F-ILDs) populations, demonstrating a correlation between increased CTGF levels and decreased pulmonary function across IPF, iNSIP, CTD-ILD, and sarcoidosis subgroups.

Our results indicate that both FVC and DLCO were significantly reduced in F-ILDs patients at the 12-month follow-up, with the decline in pulmonary function primarily attributable to the IPF subgroup. No significant reductions were observed in the iNSIP, CTD-ILD, sarcoidosis, or silicosis subgroups. Nonetheless, regression analysis revealed significant correlations between FVC and DLCO in the IPF and iNSIP subgroups.

In post-COVID-19 patients, pulmonary function tests showed improvement after 12 months, accompanied by an increase in CTGF levels. However, no significant correlation was observed between improvements in FVC and/or DLCO and the increase in CTGF levels. Radiological patterns in this group were consistent with fibrosis-like changes. All patients were receiving steroid treatment, and the relatively short time since diagnosis may explain these findings. The radiological patterns (fibrotic-like changes) and observed increase in CTGF levels underscores the importance of ongoing monitoring in post-COVID-19 patients.

Our study has certain limitations. The study duration was limited to 12 months, and long-term dynamic evaluations were not performed but are necessary. Additionally, the study was conducted on a Georgian population, which may limit the broader application of the findings to other populations. This article focuses on the correlation between CTGF and pulmonary function. The study is ongoing, and a subsequent article will address pulmonary function alterations and HRCT scan data.

## Conclusions

Our data suggest a correlation between decreased pulmonary function and increased CTGF levels in patients with IPF and other F-ILD conditions. Consequently, CTGF emerges as a potential surrogate marker for fibrosis progression in these patients. In contrast, post-COVID-19 fibrosis appears to be a non-progressive fibrotic disease; however, further monitoring is necessary.
